# Altitudinal Patterns of Diversity, Stability, and Niche Breadth in Faba Bean Rhizosphere Bacterial Communities on the Qinghai Plateau

**DOI:** 10.3390/microorganisms14091901

**Published:** 2026-08-27

**Authors:** Huilin Yan, Panda Ye, Huigang Pei, Jiajia He, Yujiao Liu

**Affiliations:** 1 College of Forestry and Grassland Science, Qinghai University, Xining 810016, China; 2023990053@qhu.edu.cn; 2 College of Agriculture and Animal Husbandry, Qinghai University, Xining 810016, China; 18798483627@163.com (P.Y.); 15544027138@163.com (H.P.); hejia_959@163.com (J.H.); 3 Academy of Agriculture and Forestry Sciences, Qinghai University, Xining 810016, China

**Keywords:** altitudinal gradient, Faba bean, rhizosphere bacteria, community stability, niche breadth

## Abstract

This study characterized regional variation in faba bean rhizosphere bacterial communities across a broad survey of the Qinghai Plateau and examined their associations with soil and spatial factors. Rhizosphere soil samples were collected from 15 faba bean-growing field sites across Haidong City, Xining City, and Hainan Tibetan Autonomous Prefecture, Qinghai Province, China, spanning elevations from 1842.9 to 3206.3 m. Nine within-site subsamples were collected at each field site, with the field site treated as the independent unit of replication. Soil physicochemical properties were measured, and bacterial communities were characterized by high-throughput sequencing of the 16S rRNA gene. At the site level, bacterial alpha-diversity indices showed numerical variation among the three regional elevation groups, but did not differ significantly. Bacterial community composition differed among groups in the site-level PERMANOVA based on Bray–Curtis dissimilarities (pseudo-F_2.12_ = 1.789, R^2^ = 0.230, *p* = 0.031), although substantial overlap among sites remained. However, a conservative locality-level sensitivity analysis was not significant (*p* = 0.060). Geographic distance was significantly associated with bacterial community dissimilarity, whereas elevation difference showed no detectable independent association after controlling for geographic distance, indicating a substantial contribution of spatial structure to the observed community differentiation. Dominant phylum-level composition was broadly conserved across sites, while genus-level relative abundances showed descriptive spatial variation. Neither RAVD-based compositional consistency nor community-level niche breadth differed significantly among groups. Among the measured soil properties, only pH differed significantly, whereas most nutrient variables did not. Overall, the study revealed spatially structured regional differentiation in faba bean rhizosphere bacterial communities across the Qinghai Plateau. The observed patterns likely reflect the combined influence of geographic structure, elevation, background soil conditions, agricultural management, plant phenology, and other unmeasured environmental factors, which could not be independently disentangled in the present survey.

## 1. Introduction

The Qinghai–Tibet Plateau is an ecologically fragile region that is highly sensitive to global climate change [1]. Owing to its high elevation, low temperatures, intense solar radiation, and short growing season, agricultural ecosystems in this region face environmental constraints and resource limitations that differ substantially from those in low-altitude areas [2]. Faba bean (*Vicia faba* L.) is an important food and forage legume in Qinghai Province, with considerable economic value and ecological functions [3]. Through symbiotic associations with rhizobia, faba bean can fix atmospheric nitrogen, while its rhizosphere microorganisms play important roles in organic matter decomposition, nutrient transformation, plant nutrient acquisition, and environmental adaptation [4]. The rhizosphere represents a highly active microecological interface where plant roots, soil, and microorganisms interact [5]. Therefore, characterizing the spatial distribution of faba bean rhizosphere bacterial communities under plateau conditions is essential for understanding the rhizosphere-associated ecological adaptation of leguminous crops in high-altitude agricultural systems.

Elevation is a major geographical factor driving environmental heterogeneity in mountain ecosystems. As elevation increases, climatic conditions such as temperature, precipitation, and solar radiation change substantially, subsequently affecting soil pH, moisture, organic carbon, and the quantity and availability of nitrogen, phosphorus, and potassium [6]. Elevational variation may also alter vegetation composition, litter inputs, plant growth, and root exudation, thereby modifying microbial habitats and resource availability [7]. Thus, elevation may not regulate microbial communities through a single direct pathway but rather through the combined effects of climate, soil properties, and plant-related factors, ultimately influencing microbial diversity, community composition, and ecological strategies.

In recent years, the distribution of soil microbial communities along elevational gradients has become an important topic in microbial ecology and biogeography. However, microbial responses to elevation vary considerably among ecosystems. In an alpine meadow in Damxung, Tibet, elevational variation in soil microbial diversity was primarily associated with soil moisture and annual precipitation [8]. Research conducted in the eastern Qinghai–Tibet Plateau showed that microbial alpha diversity decreased with increasing elevation in alpine grasslands but increased in alpine deserts, with the dominant environmental drivers also differing between the two ecosystems [9]. Along an elevational gradient in the Qilian Mountains, soil bacterial alpha diversity increased with elevation, whereas fungal diversity peaked at intermediate elevations [10]. Similar inconsistencies have been reported in other mountain ecosystems. For example, soil fungal diversity continuously decreased with increasing elevation in the Italian Alps [11], whereas soil bacterial diversity on Hallasan Mountain, Korea, exhibited a U-shaped pattern, with the lowest diversity at intermediate elevations [12]. Elevation can also alter the relative abundance of dominant microbial taxa. A study of the rhizosphere microbiome of Trillium govanianum in the Himalayas found that the relative abundance of Proteobacteria increased with elevation, whereas Actinobacteriota decreased [13]. Collectively, these findings indicate that elevational patterns of microbial communities are strongly region- and ecosystem-dependent and are jointly associated with climate, soil conditions, vegetation characteristics, and spatial processes.

In addition to community diversity and composition, community stability and niche breadth are important ecological indicators of microbial responses and adaptation to environmental variation [14]. Community stability reflects the ability of microbial communities to resist environmental disturbances and maintain relatively consistent community composition, whereas niche breadth represents the range of environmental conditions and resources used by microbial taxa [15]. High-altitude environments are typically characterized by low temperatures, reduced nutrient availability, and pronounced environmental constraints, which may strengthen environmental filtering and favor stress-tolerant microbial taxa. Consequently, high-altitude microbial communities may exhibit reduced diversity, narrower niche breadths, and altered stability [16]. Nevertheless, relatively little is known about the stability and niche characteristics of rhizosphere bacterial communities along elevational gradients in agricultural ecosystems, particularly those associated with leguminous crops on the Qinghai–Tibet Plateau.

Previous studies of faba bean in Qinghai Province have mainly focused on germplasm resources, agronomic traits, yield formation, and soil nutrient management. By contrast, the diversity, composition, stability, and niche characteristics of faba bean rhizosphere bacterial communities under different elevational conditions remain insufficiently understood [17,18]. Unlike natural grassland and forest ecosystems, agricultural ecosystems are simultaneously influenced by crop identity, agricultural management, and soil nutrient conditions, and their microbial communities may therefore respond differently to elevational variation [19]. Elucidating the elevational differentiation of rhizosphere bacterial communities in faba bean-growing areas of Qinghai can thus extend our understanding of microbial biogeographic patterns in plateau agricultural ecosystems and provide insight into the rhizosphere-associated ecological adaptation of high-altitude leguminous crops.

In this study, 15 faba bean-growing sites were selected across Haidong City, Xining City, and Hainan Tibetan Autonomous Prefecture in Qinghai Province, China. The sampling region spanned 36.21–36.97° N and 100.12–102.97° E, with elevations ranging from 1842.9 to 3206.3 m. Rhizosphere soil physicochemical properties were measured, and high-throughput sequencing of the bacterial 16S rRNA gene was used to systematically characterize bacterial diversity, community composition, community stability, and niche breadth across the elevational gradient. Soil environmental factors associated with bacterial community variation were also identified. Specifically, this study addressed the following questions: (1) Do the diversity and composition of faba bean rhizosphere bacterial communities vary significantly among altitudinal groups? (2) Do bacterial community stability and niche breadth differ across the elevational gradient? (3) Which soil physicochemical factors are most closely associated with the elevational differentiation of bacterial communities? The results provide a theoretical basis for understanding the spatial distribution and ecological adaptation of faba bean rhizosphere bacterial communities on the Qinghai Plateau.

## 2. Materials and Methods

### 2.1. Study Area Overview and Sampling

The study area encompassed 15 representative faba bean-growing sites across Haidong City, Xining City, and Hainan Tibetan Autonomous Prefecture in Qinghai Province, China (Table 1). The sampling region extended from 36.21° to 36.97° N and from 100.12° to 102.97° E, with elevations ranging from 1842.9 to 3206.3 m. Field sampling was conducted on 25 July 2024. Based on elevation, the sampling sites were classified into three groups at approximately 500 m intervals: low altitude (LF, 1800–2300 m), middle altitude (MF, 2300–2800 m), and high altitude (HF, 2800–3300 m). A total of 135 samples were collected, with 45 samples assigned to each elevation group.

All sites were sampled on 25 July 2024. At the time of sampling, faba bean plants at the LF and MF sites were generally at the pod-setting stage, whereas plants at the HF sites had completed flowering and were at the early pod-setting stage. Thus, plants across all sites were within the reproductive phase, although slight differences in developmental progression occurred among elevation groups. At each of the 15 field sites, nine spatially distributed 1 m × 1 m quadrats were established in faba bean fields. The nine quadrats within each field site represented within-site subsampling units, whereas the field site was regarded as the independent unit of replication for elevation-level inference, resulting in five independent sites per elevation group.

For soil physicochemical analyses, five soil cores were collected from the 0–15 cm layer within each quadrat using a soil auger with an internal diameter of 50 mm. The five soil cores were thoroughly homogenized to form one composite soil sample for each quadrat. For rhizosphere soil collection, surface litter was first removed, and 10 faba bean plants were randomly selected within each quadrat and carefully excavated using a sterile stainless-steel shovel, with their root systems kept as intact as possible. Surface litter was first removed, and the faba bean plants were carefully excavated with their root systems kept as intact as possible. Loosely adhering soil was removed by gentle shaking. Soil remaining tightly attached to the root surface within approximately 0–2 mm was then collected using a sterile soft-bristled brush and defined as rhizosphere soil. Rhizosphere soil collected from the 10 plants within the same quadrat was pooled and thoroughly homogenized to form one composite sample before subsampling for DNA extraction. Rhizosphere soil collected from the 10 plants within each quadrat was pooled and thoroughly homogenized to form one composite rhizosphere soil sample. All samples were immediately placed in sterile containers, transported to the laboratory under cooled conditions, and processed for soil physicochemical measurements and microbial community analyses. In total, 135 within-site subsamples were obtained from the 15 field sites. The composite soil samples collected from the 0–15 cm layer were used for physicochemical analyses and represented the bulk-soil edaphic conditions of each field site, whereas root-adhering soil within approximately 0–2 mm of the root surface was used exclusively for bacterial community sequencing.

### 2.2. Soil Physicochemical Property Determination

Soil samples used for physicochemical analyses were air-dried, gently crushed, and passed through a 2 mm sieve, whereas subsamples used for total nutrient determination were further finely ground. Eight soil physicochemical and nutrient properties were determined, including soil organic carbon (SOC), pH, total nitrogen (TN), alkali-hydrolyzable nitrogen (AN), total phosphorus (TP), available phosphorus (AP), total potassium (TK), and available potassium (AK). Soil pH was measured potentiometrically in a soil-to-deionized-water suspension at a ratio of 1:2.5 (*w*/*v*). SOC was determined by potassium dichromate oxidation. TN was measured using the Kjeldahl digestion method, and AN was determined by the alkaline hydrolysis diffusion method. TP was determined after alkali fusion followed by molybdenum-antimony colorimetry, whereas AP was extracted with 0.5 mol L^−1^ NaHCO_3_ and quantified colorimetrically using the molybdenum-antimony method. TK was determined after alkali fusion followed by flame photometry, and AK was extracted with 1 mol L^−1^ NH_4_OAc and quantified by flame photometry [20]. The magnitude and pattern of correlations differed among LF, MF, and HF, with AN and TP showing recurrent associations with community variation across the elevation groups. These group-specific results are interpreted as exploratory association patterns rather than evidence for differences in the strength of environment–community coupling.

### 2.3. DNA Extraction, PCR Amplification and High-Throughput Sequencing

Total soil DNA was extracted using the FastDNA^®^ SPIN Kit for Soil according to the manufacturer’s instructions. The V4 region of the bacterial 16S rRNA gene was amplified using the universal primer pair 515F (5′-GTGCCAGCMGCCGCGGTAA-3′) and 806R (5′-GGACTACHVGGGTATCTAAT-3′). PCR amplification was performed in a total reaction volume of 20 μL using Pro Taq DNA polymerase. The thermal cycling conditions were as follows: initial denaturation at 95 °C for 3 min; 27 cycles of denaturation at 95 °C for 30 s, annealing at 55 °C for 30 s, and extension at 72 °C for 45 s; followed by a final extension at 72 °C for 10 min.

Amplicons that passed quality assessment were subjected to paired-end sequencing on an Illumina MiSeq PE300 platform by Majorbio Bio-Pharm Technology Co., Ltd. (Shanghai, China). Raw sequence data were processed using the UPARSE pipeline implemented in USEARCH v11. After quality filtering and dereplication, sequences were clustered into operational taxonomic units (OTUs) at a 97% sequence-identity threshold using the UPARSE algorithm [21]. Chimeric sequences were identified and discarded during the UPARSE clustering procedure. Representative OTU sequences were taxonomically assigned against the SILVA SSU 16S rRNA database, release 138, using sequence coverage and identity thresholds of 0.8 [22,23]. The resulting OTU table contained 51,828–99,506 reads per sample (mean, 74,527 reads per sample). Good’s coverage ranged from 97.09% to 99.54%, with a mean of 98.18%. The OTU abundance table was subsequently used for downstream community analyses. To further examine the site-level distribution of the taxon as Rhizobium, OTUs assigned by SILVA to the genus-level group Allorhizobium–Neorhizobium–Pararhizobium–Rhizobium were extracted from the OTU table. Relative abundance was first calculated independently for each sample as the summed abundance of the target OTUs divided by the total bacterial reads in that sample. The nine nested samples within each field site were then averaged to obtain one site-level value.

### 2.4. Statistical Analysis

Because the sampling design was hierarchical, samples collected within the same field site were treated as nested subsamples, whereas the field site was considered the independent unit of replication for elevation-level inference. Each site contained nine within-site subsamples, and each elevation group therefore comprised five independent sites. Soil physicochemical variables were averaged across the nine subsamples within each site before statistical testing.

Microsoft Excel 2021 was used for data organization and preliminary calculations, and statistical analyses were performed using IBM SPSS Statistics 27.0 (IBM Corp., Armonk, NY, USA). Bacterial alpha-diversity indices were calculated using Mothur. Differences in bacterial community composition among the low-, middle-, and high-altitude groups were visualized using non-metric multidimensional scaling (NMDS) based on Bray–Curtis dissimilarities, and their statistical significance was evaluated using analysis of similarities (ANOSIM) [24]. Differences among elevation groups were evaluated by one-way ANOVA followed by Tukey’s HSD test. For bacterial community analyses, sample-level OTU abundances were converted to relative abundances and averaged within each site to generate 15 site-level community profiles. Bray–Curtis dissimilarities were calculated from these profiles, and differences among elevation groups were tested using PERMANOVA with 99,999 permutations. Geographic distances among sites were calculated from latitude and longitude coordinates.

A Mantel test was used to evaluate the distance–decay relationship between geographic distance and Bray–Curtis dissimilarity, and partial Mantel tests were used to evaluate the associations of elevation and geographic distance with bacterial community dissimilarity while controlling for each other [25].

Community stability was evaluated using the reciprocal of the Average Variation Degree (RAVD) [26]. For each OTU, the absolute deviation of its abundance in each sample from the across-sample mean was standardized by its standard deviation. The AVD of each sample was calculated as the average standardized deviation across all OTUs, and RAVD was calculated as the reciprocal of AVD, with higher values indicating greater community stability [27]. To account for the hierarchical sampling design, RAVD was first calculated for each of the 135 within-site subsamples using the same procedure as in the original analysis, after which the nine RAVD values within each site were averaged to obtain one site-level value. Differences among elevation groups were then tested using the 15 independent sites (n = 5 sites per elevation group). Taxon-level niche breadth was quantified using Levins’ niche breadth index (B). The B value of each bacterial taxon was calculated across all 135 samples using the niche.width function (method = “levins”) in the spaa R package. Community-level niche breadth (Bcom) was calculated as the relative-abundance-weighted mean of taxon-level B values within each sample. For site-level statistical inference, Bcom values from the nine within-site subsamples were averaged to obtain one Bcom value for each field site.

Different lowercase letters in tables and figures indicate significant differences among elevation groups based on multiplicity-adjusted post hoc comparisons (adjusted *p* < 0.05). Where asterisks are used to denote pairwise differences, *, **, and *** indicate adjusted *p* < 0.05, *p* < 0.01, and *p* < 0.001, respectively. Tukey HSD-adjusted *p* values were used following ANOVA, whereas Holm-adjusted *p* values were used following Dunn’s tests. *p* values from prespecified global tests, including ANOVA, PERMANOVA, Mantel, and partial Mantel tests, were reported directly because they represented individual global hypotheses rather than multiple post hoc comparisons.

## 3. Results

### 3.1. Variation in Rhizosphere Soil Physicochemical Properties Across Elevational Groups

Soil physicochemical properties differed significantly among the three elevational groups (Table 2). Soil organic carbon (SOC) and total nitrogen (TN) were significantly higher in the middle- and high-altitude groups than in the low-altitude group (*p* < 0.05), with the highest values recorded in the high-altitude group (23.25 and 1.44 g kg^−1^, respectively). Alkali-hydrolyzable nitrogen (AN) peaked in the middle-altitude group at 86.55 mg kg^−1^ and was significantly higher than that in the low- and high-altitude groups (*p* < 0.05), whereas the latter two groups did not differ significantly. Available potassium (AK) and total potassium (TK) were highest in the low-altitude group and significantly lower in the middle- and high-altitude groups. Available phosphorus (AP) did not differ significantly between the low- and middle-altitude groups but decreased markedly in the high-altitude group. Total phosphorus (TP) decreased progressively with increasing elevation, from 1.10 g/kg in the low-altitude group to 0.95 and 0.83 g/kg in the middle- and high-altitude groups, respectively. Soil pH differed significantly among elevation groups (F_2.12_ = 7.781, *p* = 0.0068), with HF showing a significantly higher pH than MF, whereas LF was intermediate and did not differ significantly from either group (Table S1). Overall, the middle- and high-altitude soils contained more SOC and TN, whereas phosphorus and potassium contents were generally lower than those at low altitude.

### 3.2. Altitudinal Variation in Faba Bean Rhizosphere Bacterial Diversity and Community Structure

**Figure 1:**
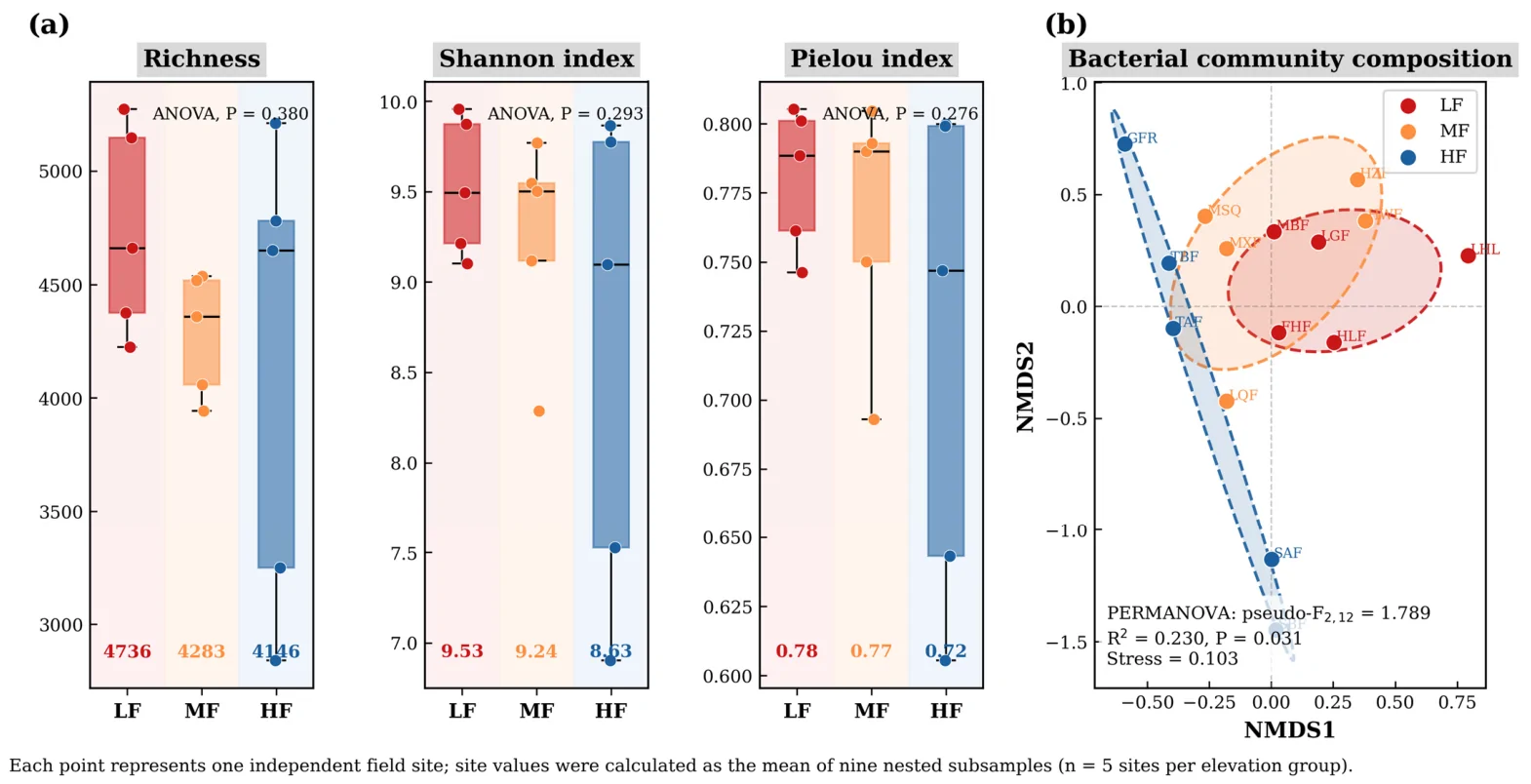
Site-level alpha and beta diversity of faba bean rhizosphere bacterial communities across elevation groups. (**a**) Alpha-diversity indices, including Richness, Shannon diversity, and Pielou evenness, calculated at the site level. Each point represents one independent field site. Nine within-site subsamples were averaged prior to site-level statistical inference (n = 5 sites per elevation group). Differences among elevation groups were evaluated using one-way ANOVA followed by Tukey’s HSD test; no significant differences were observed for the presented alpha-diversity indices (*p* > 0.05). (**b**) Non-metric multidimensional scaling (NMDS) ordination based on Bray–Curtis dissimilarities derived from 15 site-level bacterial community profiles. Dashed ellipses denote 95% confidence ellipses for each elevation group. PERMANOVA revealed significant differences in bacterial community composition across elevation groups (pseudo-F_2.12_ = 1.789, R² = 0.230, *p* = 0.031). LF, low elevation; MF, middle elevation; HF, high elevation.

Bacterial alpha diversity showed numerical variation among the three elevation groups, although no significant differences were detected at the site level (Figure 1a). The Richness index was highest in the low-elevation group (LF; 4735.82), followed by the middle-elevation (MF; 4282.91) and high-elevation groups (HF; 4148.16). A similar pattern was observed for the Shannon index, which decreased from 9.53 in LF to 9.24 in MF and 8.63 in HF. Pielou evenness also tended to decrease with increasing elevation, with values of 0.78, 0.77, and 0.72 in LF, MF, and HF, respectively. However, after accounting for the hierarchical sampling design and using field sites as the independent units of replication (n = 5 sites per elevation group), none of these alpha-diversity indices differed significantly among elevation groups (*p* > 0.05). Overall, bacterial alpha diversity exhibited a numerical tendency to decline toward higher elevations, but this pattern was not statistically significant at the site level.

At the community level, NMDS ordination based on Bray–Curtis dissimilarities showed partial differentiation among the three elevation groups, although substantial overlap remained among sites (Figure 1b). Consistent with this pattern, site-level PERMANOVA based on the 15 independent field sites revealed significant differences in bacterial community composition among elevation groups (pseudo-F_2.12_ = 1.789, R^2^ = 0.230, *p* = 0.031). Elevation group therefore explained approximately 23.0% of the variation in site-level bacterial community composition. Nevertheless, because geographic distance was also significantly associated with community dissimilarity, these differences are interpreted as elevation-associated patterns rather than as an independent effect of elevation.

### 3.3. Altitudinal Variation in Faba Bean Rhizosphere Bacterial Community Composition

**Figure 2:**
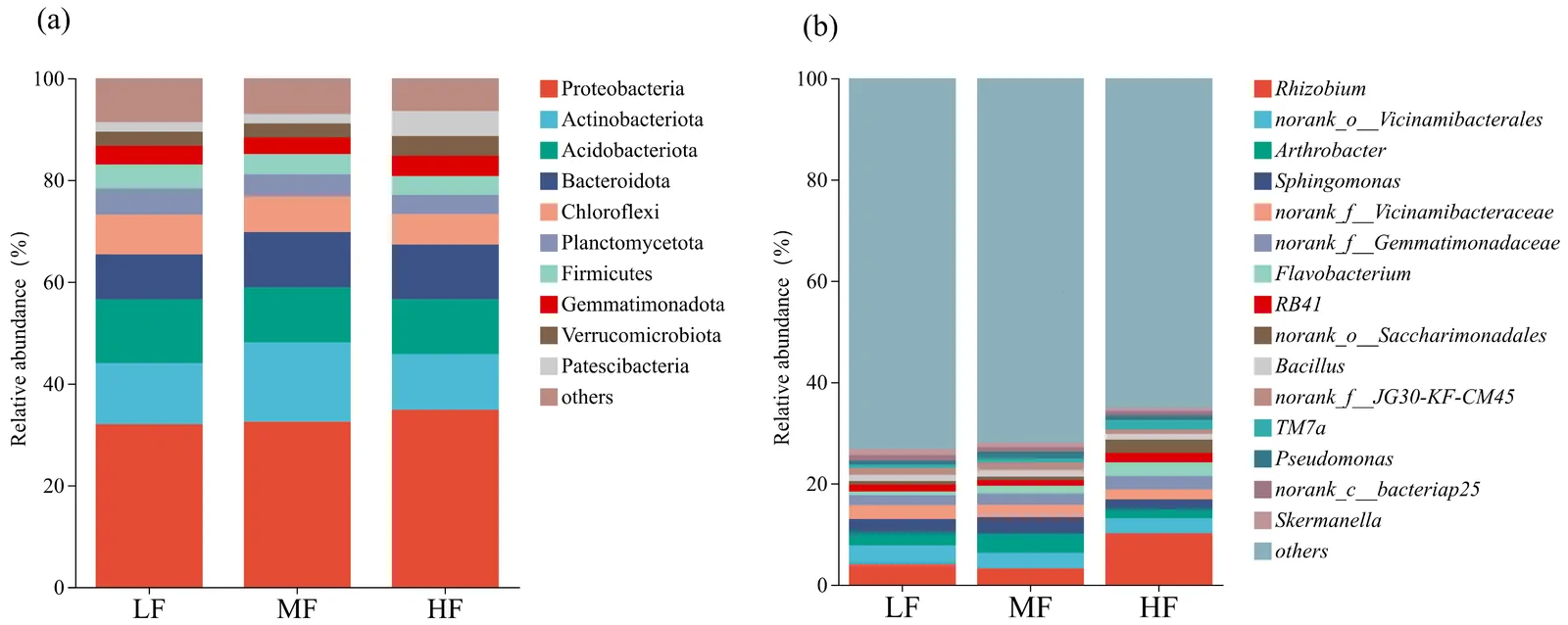
Composition of faba bean rhizosphere bacterial communities across different elevation gradients: (**a**) phylum, (**b**) genus.

Bacterial community composition showed distinct patterns among the three elevational groups at both the phylum and genus levels (Figure 2). At the phylum level, the dominant bacterial groups were generally consistent across LF, MF, and HF, with Pseudomonadota, Actinomycetota, Acidobacteriota, and Bacteroidota accounting for the largest proportions of the bacterial communities (Figure 2a). The relative abundance profiles of dominant bacterial taxa were generally similar among the three elevation groups, although numerical variation was observed in several major phyla (Figure 2a). Pseudomonadota was the most abundant phylum in all groups, accounting for 32.03%, 32.55%, and 34.87% of the bacterial communities in LF, MF, and HF, respectively. Actinomycetota showed a relatively greater proportional abundance in MF, whereas Acidobacteriota represented a smaller proportion of the community in HF. Bacteroidota showed slightly greater relative abundances in MF and HF than in LF. Overall, the dominant phylum-level composition remained broadly conserved across elevation groups, with variation mainly reflected in the relative proportions of individual phyla.

At the genus level, the SILVA-annotated *Allorhizobium–Neorhizobium–Pararhizobium–Rhizobium* group showed descriptive variation among field sites. Its mean site-level relative abundance was 3.855% in LF, 3.124% in MF, and 10.047% in HF. However, the distribution within HF was highly heterogeneous, with site-level mean relative abundances of 21.637%, 22.755%, 4.402%, 0.310%, and 1.133% at SAF, SBF, TAF, TBF, and GFR, respectively. Thus, although the taxon was detected at all five HF sites, SAF and SBF together accounted for 88.37% of the summed HF site-level signal. The group comprised 11 OTUs and was strongly dominated by OTU21444, annotated as *Rhizobium phaseoli*, which contributed 90.19% of the overall signal (Table S2).

### 3.4. Associations Between Faba Bean Rhizosphere Bacterial Communities and Soil Physicochemical Properties

**Figure 3:**
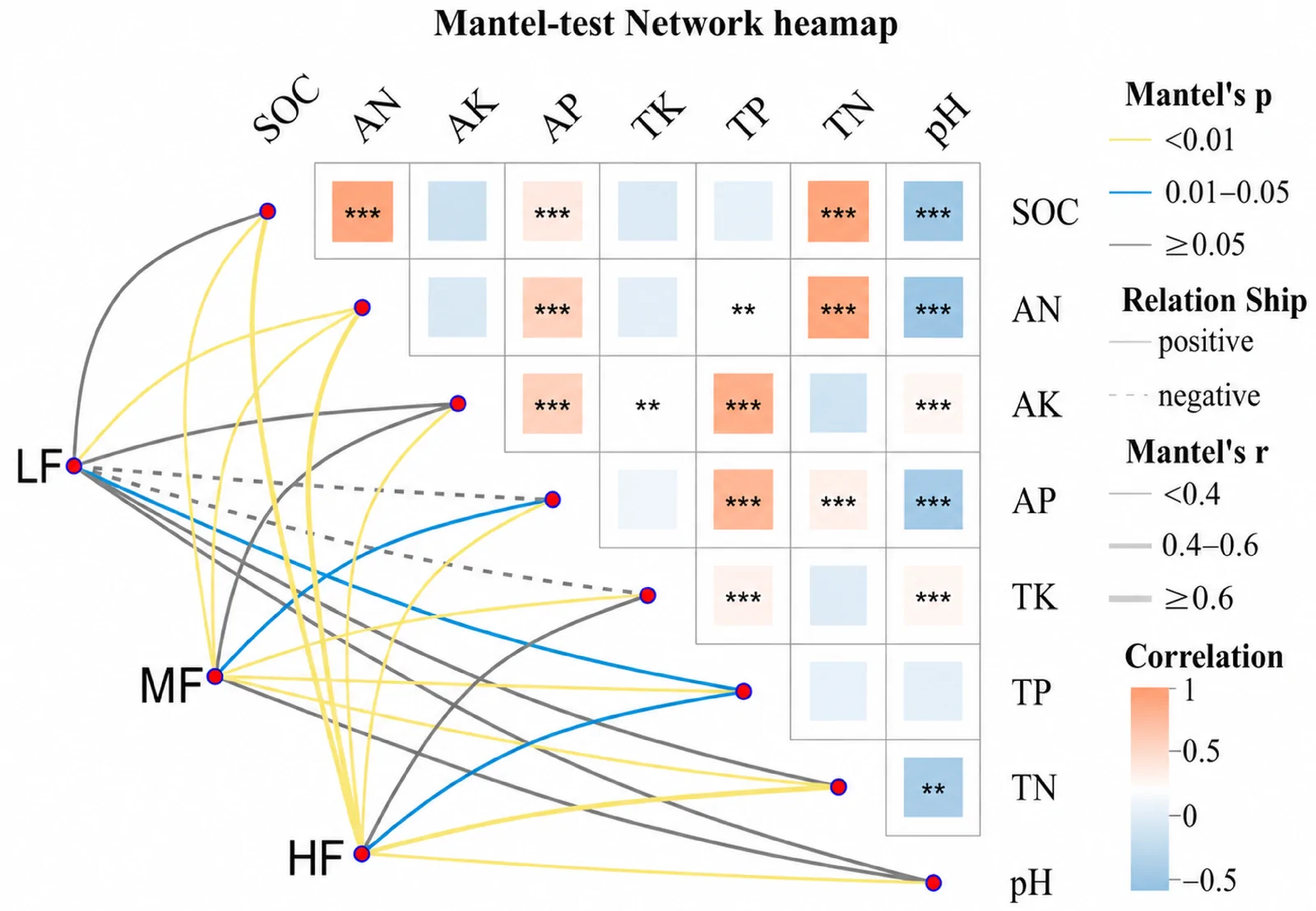
Mantel test network and correlation heatmap showing associations between faba bean rhizosphere bacterial communities and soil physicochemical properties across elevational groups. The upper-right heatmap shows Spearman correlations among soil physicochemical properties. Red and blue cells indicate positive and negative correlations, respectively, and color intensity represents the magnitude of the correlation coefficient. Asterisks indicate significant correlations: **, *p* < 0.01; and ***, *p* < 0.001. Network links represent Mantel correlations between bacterial community dissimilarity and individual soil properties within each elevational group. Yellow, blue, and gray lines indicate Mantel test probabilities of *p* < 0.01, 0.01 ≤ *p* < 0.05, and *p* ≥ 0.05, respectively. Line width represents the magnitude of Mantel’s r: <0.4, 0.4–0.6, and ≥0.6. Solid and dashed lines indicate positive and negative Mantel correlations, respectively.

Spearman correlation analysis revealed close relationships among soil carbon, nitrogen, phosphorus, and potassium properties (Figure 3). Soil organic carbon (SOC), alkali-hydrolyzable nitrogen (AN), available phosphorus (AP), and total nitrogen (TN) were significantly and positively correlated with one another. In contrast, soil pH was significantly negatively correlated with SOC, AN, AP, and TN, but positively correlated with available potassium (AK) and total potassium (TK).

Mantel associations between bacterial community composition and soil properties varied among the three elevational groups. Mantel associations between bacterial community composition and soil properties varied among the three elevation groups. In LF, significant associations were observed with AN and TP. In MF, associations were detected with SOC, AN, AP, TK, TP, and TN, whereas in HF, associations were observed with SOC, AN, AK, AP, TP, TN, and pH, with no significant association detected for TK. AN and TP were associated with bacterial community variation in all three elevation groups. These group-specific Mantel results are interpreted as descriptive association patterns rather than as evidence of differences in the strength of environment–community coupling among elevation groups.

### 3.5. Altitudinal Variation in Bacterial Community Stability

**Figure 4:**
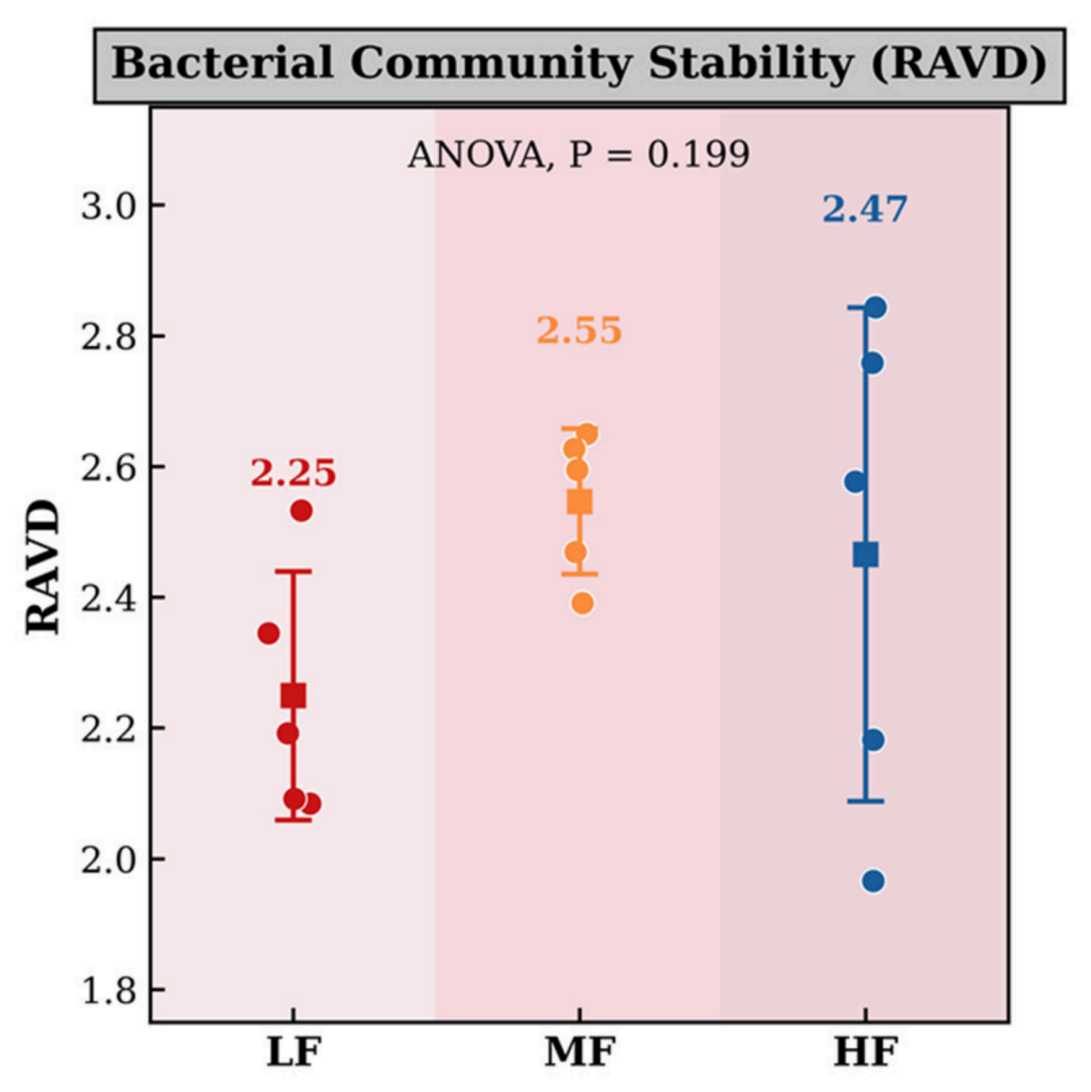
Bacterial community stability, assessed using RAVD, showed no significant differences among elevation groups at the site level. Mean RAVD values were 2.249 ± 0.190, 2.547 ± 0.111, and 2.466 ± 0.378 for the LF, MF, and HF groups, respectively (one-way ANOVA, F_2.12_ = 1.851, *p* = 0.199). Pairwise Tukey tests likewise detected no significant differences among elevation groups (all adjusted *p* > 0.19).

The stability of faba bean rhizosphere bacterial communities varied among the elevational groups, as indicated by the reciprocal of the average variation degree (RAVD) (Figure 4). At the site level, mean RAVD values were 2.249 ± 0.190, 2.547 ± 0.111, and 2.466 ± 0.378 for the LF, MF, and HF groups, respectively. Although RAVD varied numerically among elevation groups, the differences were not statistically significant when the site was treated as the independent unit of replication (one-way ANOVA, F_2.12_ = 1.851, *p* = 0.199). Pairwise Tukey tests likewise detected no significant differences among the three elevation groups (all adjusted *p* > 0.19).

### 3.6. Altitudinal Variation in Bacterial Community Niche Breadth

**Figure 5:**
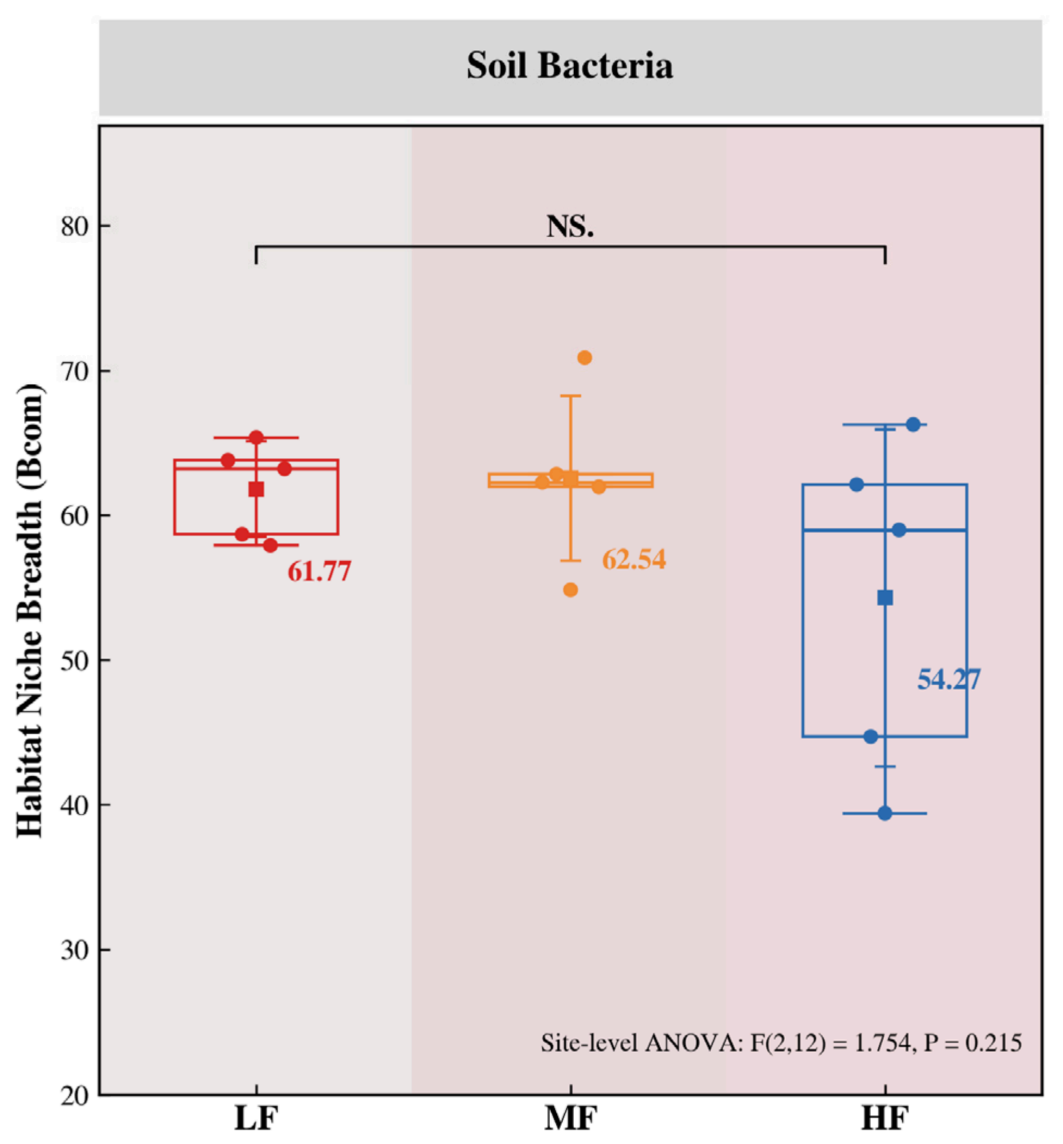
Site-level habitat niche breadth (Bcom) of faba bean rhizosphere bacterial communities across elevation groups. Taxon-level niche breadth was calculated across all 135 samples, and community-level niche breadth (Bcom) was subsequently obtained for each sample. The nine within-site subsamples were averaged to generate one site-level value for each field site, and each point in the figure represents one independent field site (n = 5 sites per elevation group). Although the HF group showed the lowest numerical Bcom, differences among elevation groups were not significant at the site level (one-way ANOVA, F_2.12_ = 1.754, *p* = 0.215). LF, low elevation; MF, middle elevation; HF, high elevation; NS, no significant difference.

Community niche breadth (Bcom) showed numerical variation among elevation groups, with the lowest mean value observed in the HF group (Figure 5). The mean Bcom values were 61.77, 62.54, and 54.27 for LF, MF, and HF, respectively. Although the HF group exhibited a lower numerical Bcom than the LF and MF groups, site-level analysis indicated that the differences among elevation groups were not statistically significant (one-way ANOVA, F_2.12_ = 1.754, *p* = 0.215). These results suggest that bacterial community niche breadth tended to be lower at high elevations, but this pattern was not significant when field sites were treated as independent replicates.

## 4. Discussion

The altitudinal differentiation of faba bean rhizosphere bacterial communities was closely associated with pronounced shifts in soil nutrient conditions. Soil organic carbon (SOC) and total nitrogen (TN) were relatively high at middle and high altitudes, whereas phosphorus and potassium contents generally declined with increasing elevation. This contrasting pattern may reflect the different responses of soil carbon–nitrogen accumulation and mineral nutrient availability to alpine environmental conditions. Environmental conditions associated with increasing elevation may have contributed to the observed changes in bacterial diversity and community structure [28]. The observed variation in soil P and K availability may reflect the combined influence of regional soil properties, parent material, and agricultural management history. Although the surveyed fields were managed under a comparable local cultivation regime, the present observational study cannot unambiguously attribute these nutrient gradients to mineral weathering alone [29]. The peak in alkali-hydrolyzable nitrogen at middle altitude may indicate that intermediate hydrothermal conditions favor organic nitrogen mineralization and maintain relatively high potential nitrogen availability [30]. Mantel tests further revealed distinct environmental association patterns among the three elevational groups. The recurrent associations of AN and TP across the three elevation groups suggest that these variables were consistently related to bacterial community variation, although the present Mantel analyses do not establish causal effects or differences in coupling strength among elevation groups [31]. An additional limitation concerns the spatial mismatch between soil physicochemical measurements and microbial sampling. Soil physicochemical properties were measured in bulk soil collected from the 0–15 cm layer, whereas bacterial communities were characterized from soil tightly adhering to the root surface. Thus, the measured soil variables represent the background edaphic conditions of each field rather than the physicochemical environment experienced directly by the rhizosphere microbiota. This distinction may be particularly relevant for available nutrients such as P, whose concentrations can differ substantially between bulk and rhizosphere soil because of plant uptake and rhizosphere processes. Consequently, the observed relationships between bulk-soil nutrient availability and rhizosphere bacterial communities should be interpreted as site-level associations rather than evidence of direct nutrient-mediated effects within the rhizosphere. Elevation should therefore be regarded as an integrated environmental gradient rather than an isolated driver, because temperature, soil moisture, plant inputs, nutrient mineralization, and pH often change simultaneously and jointly influence microbial community assembly [32].

Bacterial alpha diversity exhibited numerical variation across the elevation gradient, but the site-level reanalysis did not detect significant differences among the three elevation groups. Richness, Shannon diversity, and Pielou evenness all tended to be lower in HF than in LF. The overall decline in bacterial alpha diversity with increasing elevation is consistent with the idea that high-altitude environments impose stronger ecological filtering on rhizosphere microorganisms [33]. Reduced temperatures and lower phosphorus and potassium availability may limit microbial growth, dispersal, and resource partitioning, thereby decreasing the number of taxa able to coexist under high-altitude conditions [34]. In contrast, the relatively favorable hydrothermal environment and greater nutrient availability at low altitude may provide a wider range of ecological opportunities, supporting higher taxonomic richness, evenness, and phylogenetic diversity [35]. Although NMDS and ANOSIM analyses confirmed significant differentiation among elevational groups, the low ANOSIM R value indicated that the separation was weak. This pattern suggests that altitude-associated environmental variation influenced bacterial community composition without completely overriding the shared effects of host identity, rhizosphere selection, agricultural management, and local spatial heterogeneity.

The dominant phylum-level composition remained broadly similar among elevation groups, with Pseudomonadota, Actinomycetota, Acidobacteriota, and Bacteroidota consistently representing the major bacterial groups. Variation among elevation groups was therefore expressed mainly as changes in the relative proportions of dominant taxa rather than wholesale replacement of the major bacterial phyla [36]. The relatively high mean abundance of the SILVA-annotated Allorhizobium–Neorhizobium–Pararhizobium–Rhizobium group in HF should be interpreted cautiously. Although this taxonomic group was detected at all five HF sites, most of the HF signal was concentrated at the adjacent SAF and SBF sites, which together accounted for 88.37% of the summed HF site-level abundance. Moreover, approximately 90% of the overall signal was attributable to a single OTU (OTU21444). Therefore, the observed pattern does not provide evidence for a general enrichment of this taxonomic group at high elevation and may instead reflect local site conditions or spatially structured variation. These observations may reflect differences in rhizosphere conditions and local field environments [37]. This trend may be related to changes in rhizosphere carbon inputs and soil nitrogen status. The higher relative abundance of *Rhizobium* at high altitude may reflect the combined effects of legume rhizosphere selection and stronger plant dependence on microbially mediated nutrient acquisition under nutrient-limited conditions [38]. In contrast, the relatively high abundances of *Arthrobacter* and *Sphingomonas* at middle altitude may indicate that intermediate hydrothermal and nutrient conditions favor bacterial taxa with strong capacities for organic substrate utilization and rhizosphere adaptation [39,40]. These compositional shifts suggest that altitude altered the ecological balance among dominant bacterial genera while preserving the general phylum-level structure of the community.

The combined patterns of community stability and niche breadth further provide that environmental filtering intensified at high altitude. The high-altitude group exhibited greater compositional consistency than the middle-altitude group, as reflected by its higher reciprocal of the average variation degree, but it also had a significantly narrower community-level niche breadth than both the low- and middle-altitude groups. This combination suggests that high-altitude environments may favor a relatively restricted set of bacterial taxa with similar ecological tolerances. Strong constraints imposed by low temperature, alkaline conditions, and reduced phosphorus and potassium availability may exclude environmentally sensitive taxa and promote the persistence of specialists adapted to alpine conditions [41,42]. Broader niche breadths at low and middle altitudes may reflect greater resource heterogeneity and weaker environmental filtering, allowing generalists and specialists to coexist [36]. The absence of a significant stability difference between the low- and high-altitude groups further indicates that community consistency did not change linearly with elevation and was likely shaped by interacting effects of carbon and nitrogen accumulation, pH, nutrient availability, and local habitat heterogeneity [43].

The revised site-level analyses also changed the interpretation of community stability and niche breadth. RAVD differed numerically among elevation groups but was not significant at the site level (F_2.12_ = 1.851, *p* = 0.199). Similarly, community-level niche breadth (Bcom) was numerically lower in HF than in LF and MF, but this difference was not significant (F_2.12_ = 1.754, *p* = 0.215). In addition, Bcom was strongly related to bacterial richness, indicating that variation in niche breadth was not independent of variation in taxonomic richness. These results do not support the previous interpretation that high-elevation communities exhibited greater compositional stability, narrower ecological niches, or stronger ecological specialization. Because RAVD was calculated from samples collected during a single sampling campaign, it should be viewed primarily as an indicator of spatial compositional consistency among replicate samples rather than temporal stability. Likewise, the lower mean Bcom observed in HF represents a descriptive tendency rather than evidence of increased specialization.

Taken together, the revised analyses indicate that the most robust elevation-associated signal in this dataset lies in bacterial community composition rather than in alpha diversity, RAVD, or community niche breadth. However, community differentiation was accompanied by strong geographic structure, and several additional factors—including bulk-soil properties, agricultural management history, slight phenological differences, local habitat heterogeneity, and unmeasured climatic variation—could also have contributed to the observed patterns. Thus, the present results support the existence of spatially structured, elevation-associated variation in faba bean rhizosphere bacterial communities across the Qinghai Plateau, but they do not support a simple model in which increasing elevation alone progressively strengthens environmental filtering.

## 5. Conclusions

This study revealed clear regional and spatial differentiation in faba bean rhizosphere bacterial communities across 15 field sites on the Qinghai Plateau spanning an elevational range of 1842.9–3206.3 m. Bacterial alpha diversity showed numerical variation among groups but did not differ significantly at the site level, whereas community composition exhibited detectable regional differentiation with substantial overlap among sites. Geographic distance was significantly associated with community dissimilarity, and elevation showed no detectable independent association after accounting for geographic distance, indicating that the observed community patterns were strongly structured in space. Dominant phylum-level composition was broadly conserved, while genus-level relative abundances showed descriptive variation among sites. Neither RAVD-based compositional consistency nor community-level niche breadth differed significantly among groups. Overall, the observed bacterial community variation should be interpreted as spatially structured regional differentiation across a survey that also spans a broad elevational range, rather than as an independent effect of elevation. Geographic structure, elevation, background soil conditions, agricultural management, plant phenology, and other unmeasured environmental factors likely covaried across the survey and could not be fully disentangled. Future studies incorporating spatially balanced sampling, repeated seasonal observations, rhizosphere-specific soil chemistry, climatic measurements, and functional analyses will be necessary to resolve the relative contributions of these factors to faba bean rhizosphere bacterial community variation on the Qinghai Plateau.

## Figures and Tables

**Table 1:** Sample information. Dominant bacterial phyla and genera represent background soil microbial characteristics of the sampling areas and were not derived from the faba bean rhizosphere sequencing dataset analyzed in this study.

Sr. No.	Site Code	Longitude	Latitude	Altitude (m)	Group	Dominant Bacterial Phylum	Dominant Bacterial Genus	Soil Type
1	LHL	102.51083	36.44611	1842.9	LF	*Proteobacteria*	*Sphingomonas*	chestnut soil
2	LGF	102.42277	36.46750	2051.7	LF	*Proteobacteria*	*o__Vicinamibacterales*	chestnut soil
3	MBF	102.74750	36.21361	2093.4	LF	*Proteobacteria*	*o__Vicinamibacterales*	chestnut soil
4	HLF	101.98916	36.58944	2232.2	LF	*Proteobacteria*	*Rhizobium*	chestnut soil
5	FHF	101.85194	36.50888	2281.0	LF	*Proteobacteria*	*Rhizobium*	chestnut soil
6	MXF	102.56000	36.23416	2462.0	MF	*Proteobacteria*	*Arthrobacter*	chestnut soil
7	LQF	102.38250	36.32416	2522.2	MF	*Proteobacteria*	*Rhizobium*	chestnut soil
8	HZF	101.97472	36.89972	2556.3	MF	*Proteobacteria*	*o__Vicinamibacterales*	chestnut soil
9	HWF	102.00944	36.86638	2576.5	MF	*Proteobacteria*	*f__Vicinamibacteraceae*	chestnut soil
10	MSQ	102.81055	36.32833	2621.4	MF	*Proteobacteria*	*Arthrobacter*	chestnut soil
11	SAF	100.33638	36.44887	2946.5	HF	*Proteobacteria*	*Rhizobium*	chestnut soil
12	SBF	100.33638	36.44888	2950.0	HF	*Proteobacteria*	*Rhizobium*	chestnut soil
13	TAF	100.64200	36.68050	3014.3	HF	*Proteobacteria*	*o__Saccharimonadales*	chestnut soil
14	TBF	100.64333	36.68055	3010.0	HF	*Proteobacteria*	*o__Vicinamibacterales*	chestnut soil
15	GFR	101.17000	35.96694	3206.3	HF	*Proteobacteria*	*o__Vicinamibacterales*	chestnut soil

**Table 2:** Rhizosphere soil physicochemical properties across different elevational groups.

Variable	LF	MF	HF	ANOVA *p*
SOC (g/kg)	19.08 ± 3.78 ᵃ	22.57 ± 6.65 ᵃ	23.25 ± 7.90 ᵃ	0.553
AN (g/kg)	73.81 ± 20.80 ᵃ	86.55 ± 28.74 ᵃ	79.49 ± 33.48 ᵃ	0.778
AK (mg/kg)	178.51 ± 49.39 ᵃ	120.94 ± 65.83 ᵃ	118.46 ± 41.39 ᵃ	0.173
AP (mg/kg)	63.42 ± 12.96 ᵃ	64.65 ± 21.91 ᵃ	35.55 ± 25.64 ᵃ	0.081
TK (g/kg)	12.14 ± 1.46 ᵃ	11.31 ± 1.07 ᵃ	11.66 ± 0.67 ᵃ	0.516
TP (g/kg)	1.096 ± 0.115 ᵃ	1.286 ± 0.517 ᵃ	0.832 ± 0.314 ᵃ	0.169
TN (g/kg)	1.149 ± 0.184 ᵃ	1.050 ± 0.121 ᵃ	1.440 ± 0.432 ᵃ	0.114
pH	8.597 ± 0.160 ᵃᵇ	8.351 ± 0.091 ᵇ	8.739 ± 0.201 ᵃ	0.0068

Note: Values are presented as means ± SD based on five independent field sites per elevation group. The nine samples collected within each site were treated as nested subsamples and averaged before site-level statistical analysis. Different lowercase letters within a row indicate significant differences among elevation groups according to Tukey’s HSD test (*p* < 0.05).

## Data Availability

All libraries from Illumina amplicon sequencing were submitted to the ScienceDB (https://www.scidb.cn/s/u63qE3; https://www.scidb.cn/anonymous/dTYzcUUz; accessed on 23 July 2026).

## Supplementary Materials

The following supporting information can be downloaded at https://www.mdpi.com/article/10.3390/microorganisms14091901/s1. Table S1: Group-specific Mantel correlations between bacterial community composition and soil physicochemical variables across the three elevation groups; Table S2: Site-level relative abundance (%) of OTUs assigned to the SILVA *Allorhizobium–Neorhizobium–Pararhizobium–Rhizobium* group across the 15 field sites.
