# Supplementary material for: Altitudinal Patterns of Diversity, Stability, and Niche Breadth in Faba Bean Rhizosphere Bacterial Communities on the Qinghai Plateau
Source: Microorganisms. 2026 Aug 27;14(9):1901. doi: 10.3390/microorganisms14091901 (PMC13609657; doi:10.3390/microorganisms14091901)
Supplement: Supplementary file 1 [file microorganisms-14-01901-s001.zip › microorganisms-4487229-supplementary.pdf]

Table S1. Group-specific Mantel correlations between bacterial community composition and soil physicochemical variables across the three elevation groups.

Soil variable	LF Mantel r	MF Mantel r	HF Mantel r
DOC/SOC*	0.111	0.329	0.496
AN	0.230	0.348	0.394
AK	0.165	0.263	0.253
AP	0.031	0.372	0.173
TK	0.027	0.318	0.058
TP	0.260	0.390	0.146
TN	0.109	0.244	0.476
pH	0.171	0.043	0.393

Mantel correlations are presented as exploratory within-group associations and are not used to compare the strength of environment–community coupling among elevation groups.

Table S2. Site-level relative abundance (%) of OTUs assigned to the SILVA *Allorhizobium–Neorhizobium–Pararhizobium–Rhizobium* group across the 15 field sites

OTU ID	SILVA species annotation	n/135	Contribution (%)	LHL LF	LGF LF	MBF LF	HLF LF	FHF LF	MXF MF	LQF MF	HZF MF	HWF MF	MSQ MF	SAF HF	SBF HF	TAF HF	TBF HF	GFR HF
OTU21444	*Rhizobium phaseoli*	135	**90.1913**	0.4870	0.2482	0.4492	6.3318	9.1013	1.8050	9.3695	0.4903	0.3153	0.5101	**20.4871**	**21.8230**	4.0801	0.2370	1.0497

**OTU ID**	**SILVA species annotation**	**n/135**	**Contribution (%)**	**LHL LF**	**LGF LF**	**MBF LF**	**HLF LF**	**FHF LF**	**MXF MF**	**LQF MF**	**HZF MF**	**HWF MF**	**MSQ MF**	**SAF HF**	**SBF HF**	**TAF HF**	**TBF HF**	**GFR HF**
OTU14194	*Agrobacterium radiobacter*	135	7.0912	0.6509	0.3614	0.4843	0.2999	0.3313	0.3232	1.0409	0.2725	0.2232	0.1842	0.8010	0.8030	0.1581	0.0519	0.0512
OTU14533	Unclassified within target group	131	1.7055	0.0237	0.0088	0.0091	0.0170	0.0892	0.2482	0.4370	0.0712	0.0451	0.0092	0.2968	0.0398	0.1309	0.0119	0.0141
OTU22614	Unclassified within target group	107	0.4397	0.1437	0.0132	0.0187	0.0132	0.0025	0.0146	0.0404	0.0109	0.0055	0.0372	0.0159	0.0560	0.0006	0.0013	0.0006
OTU17821	*Pararhizobium giardinii*	132	0.3261	0.0227	0.0128	0.0215	0.0084	0.0131	0.0161	0.0280	0.0189	0.0118	0.0221	0.0346	0.0268	0.0234	0.0027	0.0148
OTU20629	Unclassified within target group	119	0.2306	0.0319	0.0081	0.0264	0.0105	0.0322	0.0054	0.0104	0.0092	0.0220	0.0219	0.0003	0.0023	0.0079	0.0048	0.0029
OTU24873	Unclassified within target group	21	0.0087	0	0	0	0.0009	0.0009	0	0.0013	0	0	0	0.0012	0.0024	0.0006	0	0
OTU21003	Unclassified within target group	12	0.0040	0.0001	0	0	0.0018	0.0009	0.0003	0.0001	0.0002	0	0	0	0	0	0	0

OTU ID	SILVA species annotation	n/135	Contribution (%)	LHL LF	LGF LF	MBF LF	HLF LF	FHF LF	MXF MF	LQF MF	HZF MF	HWF MF	MSQ MF	SAF HF	SBF HF	TAF HF	TBF HF	GFR HF
OTU24256	Unclassified within target group	8	0.0024	0	0	0	0	0	0	0.0001	0	0	0	0.0005	0.0015	0	0	0
OTU11816	*Rhizobium* sp. Y21	1	0.0003	0	0.0002	0	0	0	0	0	0	0	0	0	0	0	0	0
OTU11885	*Rhizobium* sp. Y21	1	0.0003	0	0.0002	0	0	0	0	0	0	0	0	0	0	0	0	0
**Total target group**	—	—	**100.0000**	**1.3601**	**0.6530**	**1.0092**	**6.6835**	**9.5714**	**2.4128**	**10.9277**	**0.8731**	**0.6228**	**0.7847**	**21.6374**	**22.7548**	**4.4018**	**0.3095**	**1.1333**
